# Co-generation of ethanol and l-lactic acid from corn stalk under a hybrid process

**DOI:** 10.1186/s13068-018-1330-6

**Published:** 2018-12-18

**Authors:** Yong Wang, Jinlong Liu, Di Cai, Guoqun Zhao

**Affiliations:** 1 Fermentation Engineering Technology Research Center of Heibei Province, College of Bioscience & Bioengineering, Hebei University of Science and Technology, Shijiazhuang, 050000 People’s Republic of China; 2 National Energy R&D Center for Biorefinery, Beijing University of Chemical Technology, Beijing, 100029 People’s Republic of China

**Keywords:** Corn stover, Ethanol, l-Lactic acid, Co-generation, *Saccharomyces cerevisiae*, *Bacillus coagulans*

## Abstract

**Background:**

Corn stover, as one important lignocellulosic material, has characteristics of low price, abundant output and easy availability. Using corn stover as carbon source in the fermentation of valuable organic chemicals contributes to reducing the negative environmental problems and the cost of production. In ethanol fermentation based on the hydrolysate of corn stover, the conversion rate of fermentable sugars is at a low level because the native *S. cerevisiae* does not utilize xylose. In order to increase the conversion rate of fermentable sugars deriving from corn stover, an effective and energy saving biochemical process was developed in this study and the residual xylose after ethanol fermentation was further converted to l-lactic acid.

**Results:**

In the hybrid process based on the hydrolysate of corn stover, the ethanol concentration and productivity reached 50.50 g L^−1^ and 1.84 g L^−1^ h^−1^, respectively, and the yield of ethanol was 0.46 g g^−1^. The following fermentation of l-lactic acid provided a product titer of 21.50 g L^−1^ with a productivity of 2.08 g L^−1^ h^−1^, and the yield of l-lactic acid was 0.76 g g^−1^. By adopting a blank aeration before the inoculation of *B. coagulans* LA1507 and reducing the final cell density, the l-lactic acid titer and yield reached 24.25 g L^−1^ and 0.86 g g^−1^, respectively, with a productivity of 1.96 g L^−1^ h^−1^.

**Conclusions:**

In this work, the air pumped into the fermentor was used as both the carrier gas for single-pass gas stripping of ethanol and the oxygen provider for the aerobic growth of *B. coagulans* LA1507. Ethanol was effectively separated from the fermentation broth, while the residual medium containing xylose was reused for l-lactic acid production. As an energy-saving and environmental-friendly process, it introduced a potential way to produce bioproducts under the concept of biorefinery, while making full use of the hydrolysate of corn stover.

## Background

In ethanol production, lignocellulosic biomass which is the most abundant raw material has been widely used [1]. Due to its projected positive attributes in terms of economic, environmental, and social sustainability, cellulosic ethanol has been widely regarded as a promising alternative liquid fuel [2]. However, the cellulosic ethanol production is extremely limited because the dominant pentose sugar in hydrolysate of lignocellulosic biomass can not be utilized by the native *S. cerevisiae* [3]. Thus, the hydrolysates obtained from lignocellulosic biomass containing xylose require an economic conversion in biorefinery process through xylose utilization.

It is a challenge to directly construct genetically engineered *S. cerevisiae* that would be able to ferment xylose in lignocellulose hydrolysates to ethanol [4]. The introduction into *S. cerevisiae* of a pathway for xylose-utilizing organism has been intensively studied [5–9]. However, due to internal limitations of engineered *S. cerevisiae*, multifarious optimization was normally required [10]: modified genes coding for enzymes in the pentose phosphate pathway [11], random mutagenesis [12] and laboratory evolution [12, 13] have been employed to further improve xylose-fermenting ability.

To effectively utilize xylose, the production of high-value products such as furfural and xylitol along with ethanol fermentation based on cellulose under the biorefinery concept has been reported [14, 15]; however, a relatively small market limits the development of above products [16]. Other than a single-organism approach, a new strategy for efficient co-utilization of glucose and xylose from the hydrolysates of corn stalk was proposed in this study, and a two-stage fermentation was conducted for cogeneration of ethanol and l-lactic acid.

## Results

### Products accumulation and sugars utilization during ethanol and l-lactic acid fermentation

**Fig. 1:**
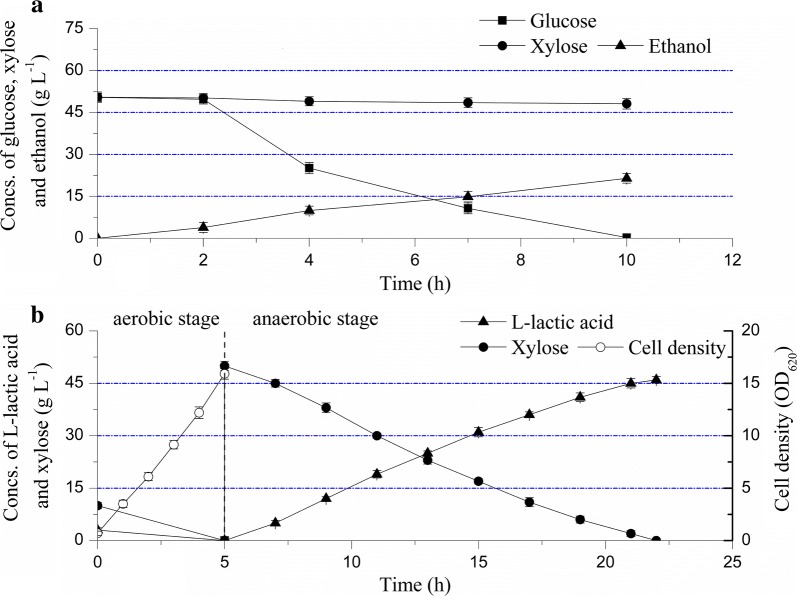
Sugars utilization and products accumulation in **a** ethanol and **b** l-lactic acid fermentation. The error bars in the figure indicate the standard deviations of three parallel replicates

For *S. cerevisiae* M3013 and *B. coagulans* LA1507, to investigate the utilization capacity of carbon sources, batch fermentations were conducted as shown in Fig. 1. During ethanol fermentation, glucose was rapidly exhausted within 10.00 h and no xylose uptake was detected (Fig. 1a). The ethanol titer and productivity reached 21.45 g L^−1^ and 2.15 g L^−1^ h^−1^, respectively, and a yield of 0.47 g g^−1^ was obtained. The xylose metabolism defect of native *S. cerevisiae* has been reported by Barnett in 1976 [17], which was confirmed in *S. cerevisiae* M3013. Profiles of sugars utilization and l-lactic acid production by *B. coagulans* LA1507 Are illustrated in Fig. 1b. In aerobic stage, a cell density (OD_620_) of 15.90 was eventually obtained, and 10.00 g L^−1^ xylose was totally consumed within 5.00 h. No accumulation was observed in aerobic stage, indicating that l-lactic acid synthesis was down-regulated in favor of efficient proliferation and maintenance of cells under the stress condition of oxygen. In the anaerobic stage, an efficient conversion of xylose into l-lactic acid was observed from 5.00 to 22.00 h, and the rates of xylose consumption and l-lactic acid accumulation were nearly constant. The results indicated that no carbon catabolite repression (CCR) worked on *B. coagulans* when the xylose concentration reached 50 g L^−1^. In agreement with the previous study, a high yield of l-lactic acid with xylose (0.99 g g^−1^) was obtained [18], and l-lactic acid titer and productivity reached 46.00 g L^−1^ and 2.71 g L^−1^ h^−1^, respectively. Interestingly, the complementary utilization on carbon sources of strain M3013 and LA1507 introduces an effective way to cogenerate ethanol and l-lactic acid based on corn stalk.

### The dynamics in gas stripping of ethanol and the effect of ethanol on the cell growth of *B. coagulans* LA1507

**Fig. 2:**
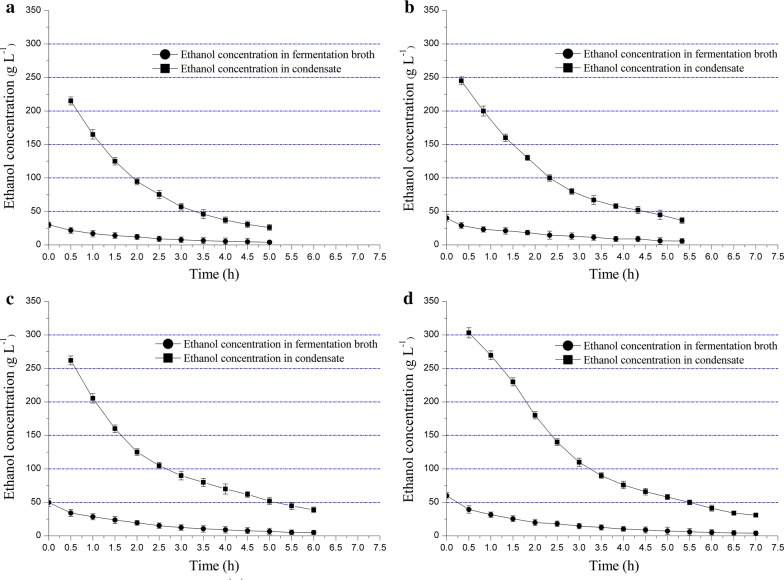
Kinetic curves of gas stripping of ethanol at different initial ethanol concentrations: **a** 30.00 g L^−1^, **b** 40.00 g L^−1^, **c** 50.00 g L^−1^, **d** 60.00 g L^−1^. The error bars in the figure indicate the standard deviations of three parallel replicates

Kinetic analysis was conducted in the gas stripping of ethanol, and initial ethanol concentrations ranging from 30.00 to 60.00 g L^−1^ were adopted. As shown in Fig. 2a, when the initial ethanol concentration was 30.00 g L^−1^, the ethanol content in fermentation broth continuously decreased to 4.00 g L^−1^, and the ethanol concentration in condensate decreased from 215.00 to 26.00 g L^−1^ within 5.00-h aeration. When the initial ethanol concentration increased to 40.00 g L^−1^, 5.85 g L^−1^ ethanol was achieved in fermentation broth after 5.33-h aeration during which the ethanol content in condensate decreased from 245.00 to 36.60 g L^−1^ (Fig. 2b). In addition, the initial ethanol concentration in condensate reached 262.00 g L^−1^ based on the fermentation broth containing 50.00 g L^−1^ ethanol before aeration, and the ethanol content in condensate and fermentation broth decreased to 38.90 g L^−1^ and 5.00 g L^−1^, respectively (Fig. 2c). Finally, when the initial ethanol concentration was set as 60.00 g L^−1^, a maximum concentration of ethanol in condensate (303.00 g L^−1^) was obtained, and the ethanol content in condensate and fermentation broth decreased to 31.00 g L^−1^ and 4.00 g L^−1^, respectively (Fig. 2d). To sum up, the results showed that the initial ethanol content in condensate increased with the rise of ethanol content in fermentor, and the downward trend of ethanol concentration in fermentor slowed down when the ethanol content approximately reached 5.00 g L^−1^.

**Fig. 3:**
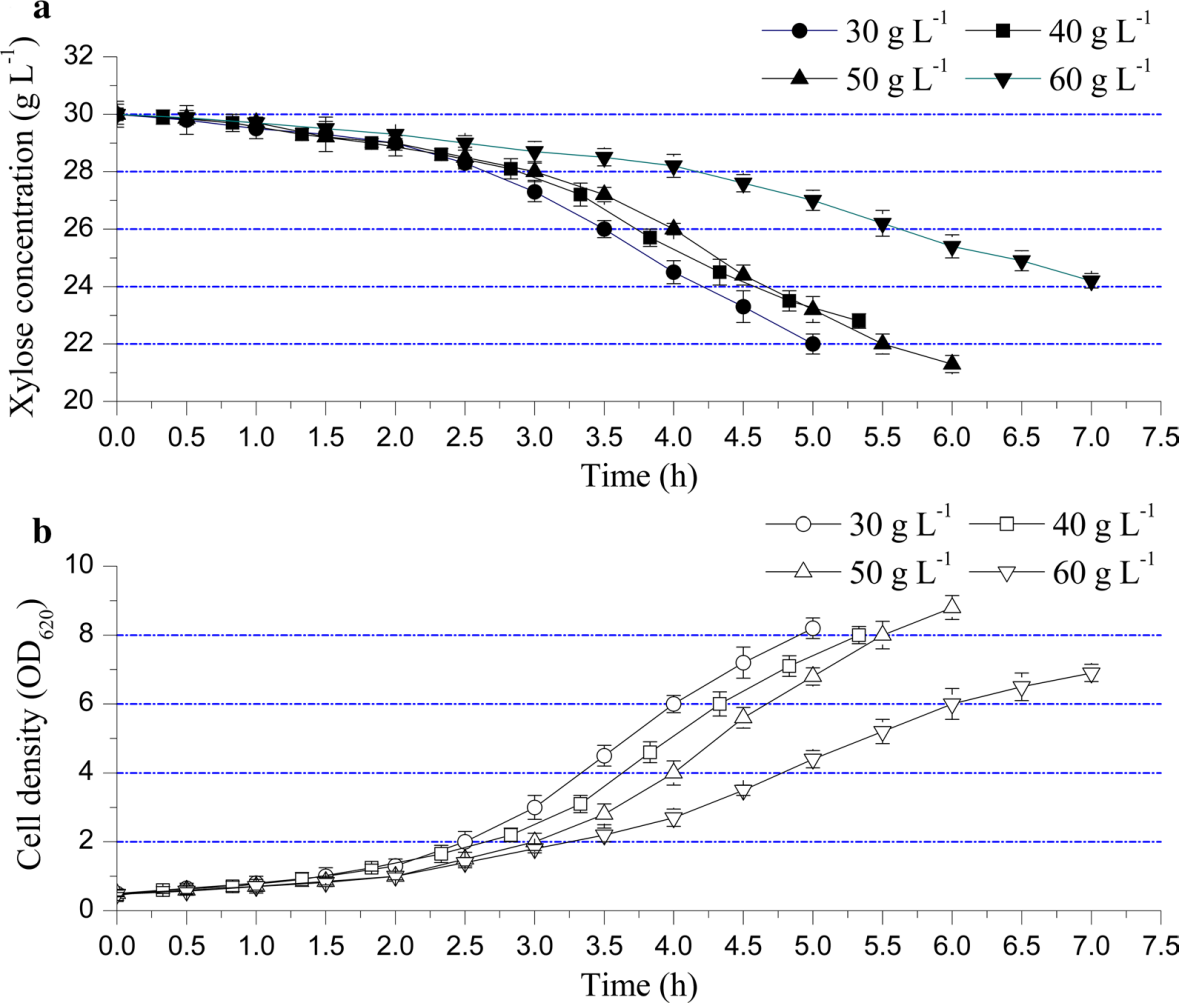
The cell growth of *B. coagulans* LA1507 when different initial concentrations of ethanol were adopted. The error bars in the figure indicate the standard deviations of three parallel replicates

To reduce the energy consumption of ethanol separation, the gas stripping of ethanol and aerobic culture of *B. coagulans* LA1507 were simultaneously conducted. As reported by Alzate et al., when the ethanol concentration was about 60.00 g L^−1^, the unit energy costs were energy intensive as it would require 27.89 MJ L^−1^ ethanol in distillation step [19]. In this study, when the initial ethanol concentration was 50.00 g L^−1^, the integrated value of ethanol content in the condensate could reach about 200.00 g L^−1^ after gas stripping. The increase in ethanol concentration (200.00 g L^−1^ vs. 60.00 g L^−1^) before distillation should reduce about 70.00% of the energy (19.52 MJ L^−1^) required for further purification. In addition, the effect of ethanol concentration on the cell growth of *B. coagulans* LA1507 was also studied as shown in Fig. 3. When the initial ethanol concentration was 30.00 g L^−1^, the xylose concentration decreased from 30.00 to 22.00 g L^−1^ during 5.00-h aeration, and the cell density (OD_620_) increased from 0.50 to 8.20. When the initial ethanol concentration increased to 40.00 g L^−1^, a final cell density (OD_620_) of 8.00 was obtained after 5.33 h, and the xylose concentration decreased from 30.00 to 22.80 g L^−1^. And 50.00 g L^−1^ ethanol was next adopted, the cell density (OD_620_) of *B. coagulans* LA1507 accumulated to 8.80 within 6.00 h, while the xylose concentration decreased from 30.00 to 21.30 g L^−1^. Although no obvious inhibition effect of ethanol was observed when its initial concentration increased from 30.00 to 50.00 g L^−1^, the aeration time slightly extended to achieve the same level of cell density (Fig. 3b). However, when the initial ethanol concentration was further increased to 60.00 g L^−1^, the cell density (OD_620_) was only 6.90 with an inefficient xylose utilization (from 30.00 to 24.20 g L^−1^) and an extended aeration time (7.00 h), indicating a significant inhibition effect of ethanol (Fig. 3b). Taken together, to balance the ethanol titer and the inhibition effect of ethanol on the cell growth of *B. coagulans* LA1507, the results indicated that the suitable level of initial ethanol concentration was 50.00 g L^−1^ in this study.

### The effect of ethanol on l-lactic acid production in anaerobic fermentation of *B. coagulans* LA1507

**Fig. 4:**
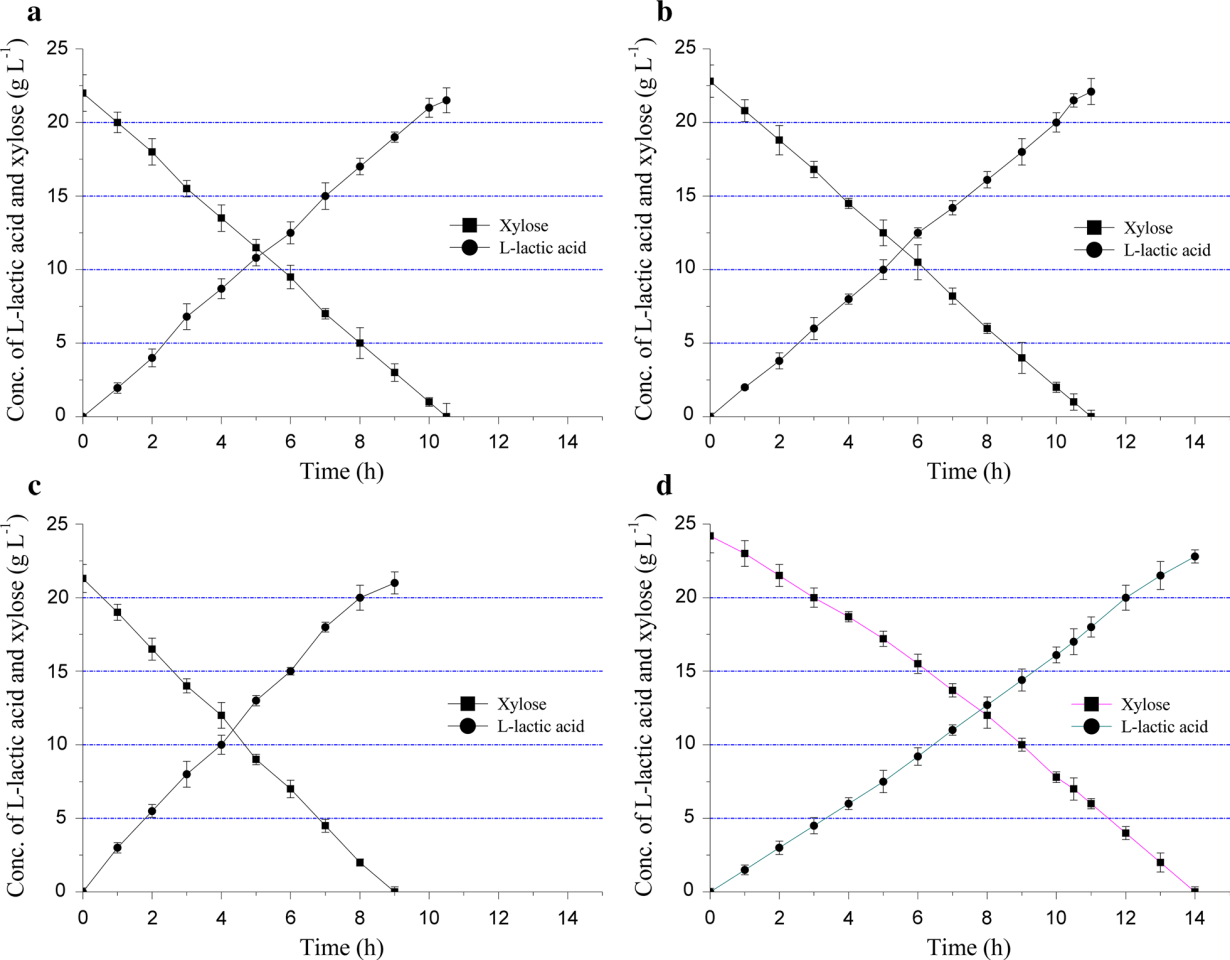
Concentration changes of l-lactic acid and xylose in the anaerobic fermentation of *B. coagulans* with different initial ethanol concentration levels: **a** 30.00 g L^−1^, **b** 40.00 g L^−1^, **c** 50.00 g L^−1^, **d** 60.00 g L^−1^. The error bars in the figure indicate the standard deviations of three parallel replicates

In the hybrid process cogenerating ethanol and l-lactic acid, the ethanol concentration affected the growth of *B. coagulans* LA1507 in the aeration stage, and the cell density of *B. coagulans* LA1507 had a further effect on l-lactic acid production. As shown in Fig. 4, l-lactic acid production was studied after the aeration stage with different initial ethanol concentrations. With an initial ethanol concentration of 30.00 g L^−1^, xylose (22.00 g L^−1^) in the fermentation broth was totally consumed accumulating 21.50 g L^−1^ l-lactic acid, and the yield and productivity reached 0.79 g g^−1^ and 2.05 g L^−1^ h^−1^, respectively (Fig. 4a). When the initial ethanol concentration was 40.00 g L^−1^, the l-lactic acid concentration reached 22.10 g L^−1^ based on the residual xylose (22.80 g L^−1^) after the aeration stage, and a yield of 0.81 g g^−1^ was obtained with a productivity of 2.01 g L^−1^ h^−1^ (Fig. 4b). The similar results obtained in l-lactic acid fermentation at 30.00 g L^−1^ and 40.00 g L^−1^ ethanol were probably caused by the almost same level of cell density (Table 1). When the ethanol concentration increased to 50.00 g L^−1^, the l-lactic acid titer was 21.00 g L^−1^, and a maximum of cell density (an OD_620_ of 8.80) was obtained which promoted the increase of productivity (2.33 g L^−1^ h^−1^); however, more carbon source was utilized for cell growth, resulting in the decrease of l-lactic acid yield (0.77 g g^−1^) (Fig. 4c). Finally, when the initial ethanol concentration was 60.00 g L^−1^, the l-lactic acid titer reached 22.80 g L^−1^ within an obviously extended fermentation (14.00 h), and the yield achieved 0.84 g g^−1^; particularly, a relative low cell density (OD_620_) was obtained under the obvious inhibition effect of ethanol (60.00 g L^−1^), which resulted in a low productivity (1.63 g L^−1^ h^−1^) (Fig. 4d). The results indicated that the maximum cell density and productivity were achieved at 50.00 g L^−1^ ethanol; thus, the ethanol concentration in further experiments was set as 50.00 g L^−1^ in this study (Table 1).

**Table 1:** Comparison of l-lactic acid fermentation characteristics based on simulated substrate and alkali-treated corn stalk (ATCS) hydrolysates

Culture	Initial ethanol concentration (g L^−1^)	Aeration time (h)	Cell density (OD_620_)	l-Lactic acid titer (g L^−1^)	l-Lactic acid yield (g g^−1^)	l-Lactic acid productivity (g L^−1^ h^−1^)
Simulated substrate	30.00	5.00	8.20	21.50	0.79	2.05
	40.00	5.33	8.00	22.10	0.81	2.01
	50.00	6.00	8.80	21.00	0.77	2.33
	60.00	7.00	6.90	22.80	0.84	1.63
ATCS hydrolysates	50.50	8.00	8.80	21.50	0.76	2.08
	50.30	6.50	6.90	24.25	0.86	1.96

### Co-generation of ethanol and l-lactic acid based on corn stalk

**Fig. 5:**
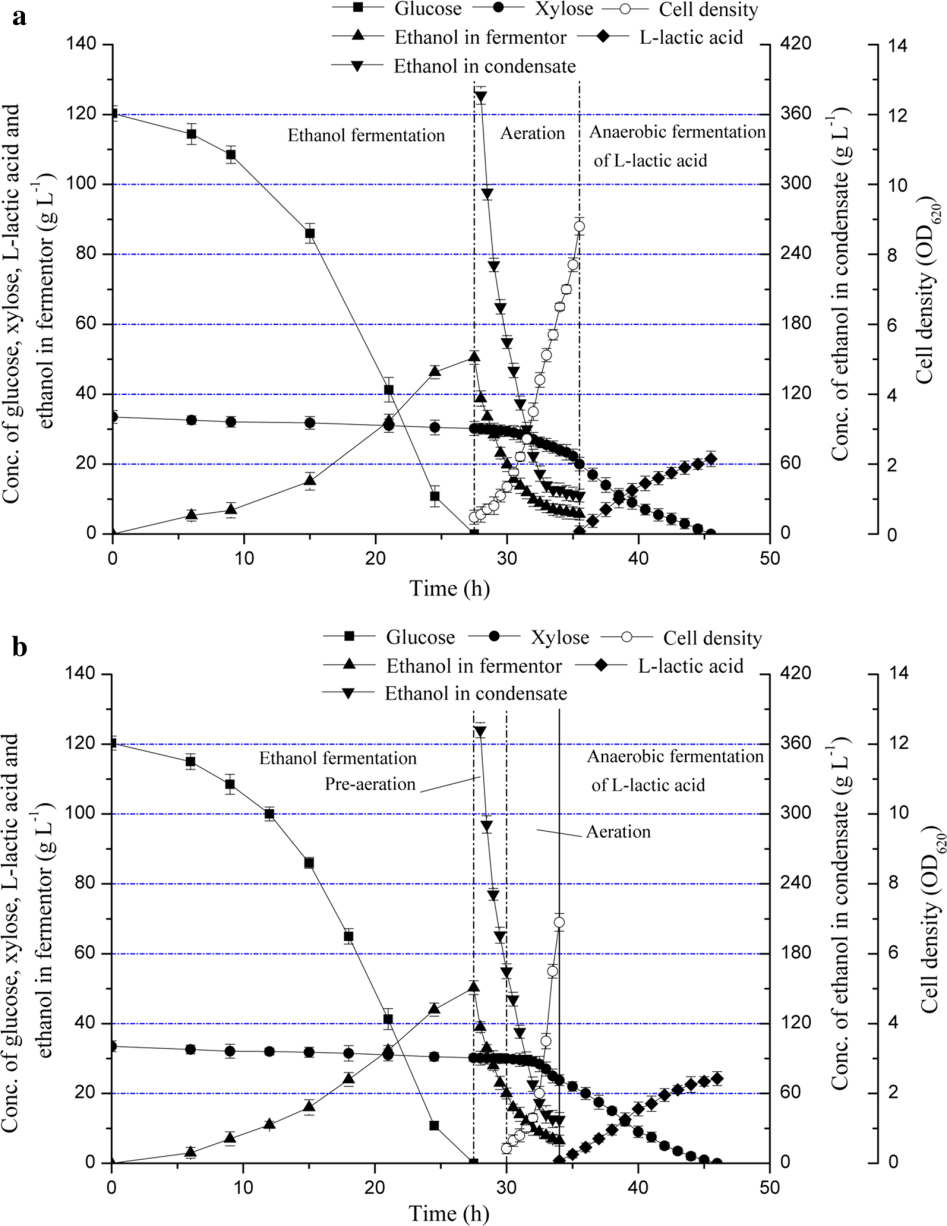
Co-generation of ethanol and l-lactic acid from corn stalk hydrolysates: **a** without pre-aeration and **b** with pre-aeration. The error bars in the figure indicate the standard deviations of three parallel replicates

Co-generation of ethanol and l-lactic acid from corn stalk was investigated, and the enzymatic hydrolysates of dry corn stalk (DCS) contained 120.30 g L^−1^ glucose and 33.50 g L^−1^ xylose. As shown in Fig. 5a, ethanol fermentation was conducted before 27.50 h with an ethanol titer of 50.50 g L^−1^, and the ethanol yield and productivity reached 0.46 g g^−1^ and 1.84 g L^−1^ h^−1^, respectively. Moreover, the dilution effect of neutralizer addition caused the slight decrease of xylose concentration (from 33.50 to 30.20 g L^−1^). The aerobic culture of *B. coagulans* LA1507 and the gas stripping of ethanol were performed simultaneously in the aeration stage (from 27.50 to 35.50 h), and the cell density (OD_620_) of *B. coagulans* LA1507 achieved 8.80 consuming 10.20 g xylose. The ethanol concentration in fermentor decreased from 50.50 to 5.70 g L^−1^ during the gas stripping process, and the ethanol content in condensate declined from 376.40 to 33.00 g L^−1^. Under the same level of ethanol content (50.00 g L^−1^), the increase of the initial ethanol concentration and the decrease of the final ethanol concentration in condensates comparing to the previous results of kinetic experiment (262.00 g L^−1^ and 38.90 g L^−1^) indicated that the composition difference of culture medium may contribute to the gas stripping of ethanol, which need to be further studied. The anaerobic fermentation of l-lactic acid was conducted from 35.50 to 45.50 h, and the l-lactic acid titer reached 21.50 g L^−1^ with a productivity of 2.08 g L^−1^ h^−1^; however, the high cell density (OD_620_ = 8.80) of *B. coagulans* LA1507 resulted in a relative low product yield (0.76 g g^−1^).

A pre-aeration process was further introduced to reduce both ethanol inhibition effect on *B. coagulans* LA1507 and the aeration time. As shown in Fig. 5b, during the ethanol fermentation (before 27.50 h), an ethanol titer of 50.30 g L^−1^ was obtained, and the ethanol yield and productivity reached 0.46 g g^−1^ and 1.83 g L^−1^ h^−1^, respectively. The pre-aeration was conducted from 27.50 to 30.00 h, and the ethanol concentration in condensate declined from 372.00 to 165.00 g L^−1^; moreover, the ethanol concentration in fermentor decreased by 60.24%, which would significantly reduce the ethanol inhibition effect on *B. coagulans* LA1507. The gas stripping of ethanol and the aerobic culture of *B. coagulans* LA1507 were simultaneously conducted from 30.00 to 34.00 h. The ethanol concentration in fermentor further decreased from 20.00 to 6.50 g L^−1^, and the final ethanol content in condensate was 37.50 g L^−1^. In addition, the cell density (OD_620_) of *B. coagulans* LA1507 increased rapidly from 0.42 to 6.90 within 4.00 h, and the relative low level of cell density contributes to the increase of l-lactic acid yield, while reducing the xylose consumption for cell growth. In l-lactic acid fermentation (from 34.00 to 46.00 h), the residual xylose was totally consumed with a product titer of 24.25 g L^−1^, and the productivity achieved 1.96 g L^−1^ h^−1^. As expected, the l-lactic acid yield increased to 0.86 g g^−1^ when the cell density was controlled at 6.90.

**Fig. 6:**
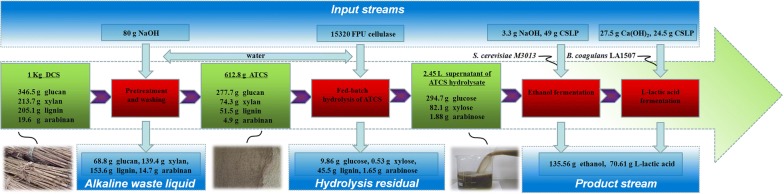
The mass balance of the experimental process based on 1.00 kg DCS

Comparing with the co-generation process without pre-aeration, when a pre-aeration was adopted, the total aeration time decreased from 8.00 to 6.50 h, and the l-lactic acid yield significantly increased from 0.76 to 0.86 g g^−1^ (Table 1). Interestingly, although the cell density was controlled at a low level with pre-aeration (OD_620_ = 6.90), the productivity obtained (1.96 g L^−1^ h^−1^) was near that of the process without pre-aeration (2.08 g L^−1^ h^−1^), indicating the reduced ethanol inhibition effect on *B. coagulans* LA1507 fermentation. A mass balance of the experiment process was evaluated, and 135.56 g ethanol and 70.61 g l-lactic acid were finally obtained from 1.00 kg DCS (Fig. 6). It is worth to mention that a considerable amount of fermentable sugars remaining in the alkaline waste liquid and the hydrolysis residual was still not utilized effectively, which needs further research.

## Discussion

Two-stage fermentation has been widely studied for the cogeneration of hydrogen and methane, and the residual H_2_-producing solution riching in volatile fatty acids has been reutilized by the methanogen community for methane production to increase the energy conversion efficiency [20–22]. In this study, a two-stage fermentation was conducted to efficiently utilize the residual xylose in ethanol-producing solution, and the feasibility of cogenerating ethanol and l-lactic acid was studied, which has been rarely reported.

The lack of xylose reductase and xylitol dehydrogenase activities in native *S. cerevisiae* leads to a defect of xylose utilization [23]. Currently, the bacterial conversion of xylose into ethanol has been studied mostly by the recombinant microorganisms; however, ethanol inhibition and fastidious requirements for aerobic condition of metabolic engineering strains limited the large-scale industrial application [24]. Thus, the efficient utilization of biomass resources by process improvement under the concept of biological refining was proposed in this study.

In agreement with the previous reports, it was confirmed that the native strain *S. cerevisiae* M3013 does not utilize xylose (Fig. 1a). Comparing with ethanol fermentations under very high gravity (VHG) conditions [25, 26], the ethanol yield and productivity obtained in this study indicated that no inhibition effect was generated with the carbon source containing 120.30 g L^−1^ glucose and 33.50 g L^−1^ xylose. In addition, the efficient utilization of xylose by *B. coagulans* LA1507 in l-lactic acid fermentation was observed (Fig. 1b). Owing to its advantageous characteristics in terms of growing and fermenting at high temperature (50.00–60.00 °C), simple nutrition requirement and high optical purity of the product [27], *B. coagulans* LA1507 was used for non-sterilized fermentation of l-lactic acid following the ethanol production. The complementary utilization in carbon source of strain M3013 and strain LA1507 provides us important insights to elucidate the mechanism of the hybrid process containing a two-stage fermentation.

A significant source of cost and energy consumption in current ethanol production is the dewatering and drying of ethanol [28]. Thus, the gas stripping of ethanol and the aerobic culture of *B. coagulans* LA1507 were simultaneously conducted in this study aiming to reduce the separation cost. According to Raoult’s law, the vapor pressure of a volatile compound in a dilute solution increased linearly with its molar concentration in the solution. In ABE separation process of butanol fermentation, Xue et al. found that the stripping rate decreased with the reduced ABE concentration in the solution and the gas stripping became ineffective when ABE concentration was below 5.00 g L^−1^ [29]. The same phenomenon was also observed in the present study, and the final ethanol concentration in fermentor after gas stripping was also near the level of 5.00 g L^−1^. In this study, under the optimized conditions, the integrated value of ethanol content in condensate reached about 200.00 g L^−1^ which was four times of ethanol titer after fermentation. The highly concentrated condensate that far exceeded ethanol titer would significantly contribute to reducing ethanol separation cost.

Lactic acid bacteria (LAB) are Gram-positive bacteria which can survive and grow in ethanol-containing environments [30]; however, the toxic effect of ethanol on cell membrane can not be ignored, which further inhibits the growth and viability of bacteria [31]. Thus, the gas stripping of ethanol was conducted to alleviate the inhibition effect on *B. coagulans* LA1507, and the results obtained imply that no significant ethanol inhibition on *B. coagulans* LA1507 growth and l-lactic acid production was generated when the initial ethanol concentration reached 50.00 g L^−1^. Moreover, as shown in Fig. 5, when a pre-aeration was introduced in the cogeneration process based on the hydrolysates of alkali-treated corn stalk (ATCS), the aeration time significantly decreased from 7.00 to 4.00 h to achieve the same level of cell density (OD_620_ = 6.90). In addition, the l-lactic acid productivity with low cell density (OD_620_ = 6.90) achieved 1.96 g L^−1^ h^−1^ which was near 2.08 g L^−1^ h^−1^ obtained with high cell density (OD_620_ = 8.80). These data imply that the gas stripping of ethanol promotes both the cell growth of *B. coagulans* LA1507 and the production of l-lactic acid.

The yeast residue was removed from the broth by centrifugation after ethanol fermentation, which facilitated the cell density determination of *B. coagulans* LA1507. However, in fact, if the cells of *S. cerevisiae* retained in the fermentation broth, the high temperature of l-lactic acid fermentation in this study could contribute to the autolysis of yeast [32]. And it has been widely reported that the yeast autolysates riching in amino acids and vitamins has a significant positive effect on lactic acid production [27, 33–35]. The utilization of yeast autolysate as nitrogen source would both reduce the raw material cost and intensify the hybrid process, which need to be further studied.

## Conclusion

In this paper, the hybrid process containing a two-stage fermentation was investigated for cogeneration of ethanol and l-lactic acid based on corn stalk. The gas stripping of ethanol and the effect of ethanol on l-lactic acid fermentation were studied, and the optimized ethanol titer was found to be 50.00 g L^−1^. Using ATCS hydrolysates as substrate, an ethanol titer of 50.30 g L^−1^ was obtained, and the yield and productivity of ethanol reached 0.46 g g^−1^ and 1.83 g L^−1^ h^−1^, respectively. In addition, the hybrid process provided a l-lactic acid titer of 24.25 g L^−1^ with a productivity of 1.96 g L^−1^ h^−1^. Comparing with the process without pre-aeration, the total aeration time decreased from 8.00 to 6.50 h and the l-lactic acid yield significantly increased from 0.76 to 0.86 g g^−1^ in the process with pre-aeration, while keeping the productivity at the same level. A mass balance of the experiment procedure was evaluated, and 135.56 g ethanol and 70.61 g l-lactic acid were finally obtained from 1.00 kg DCS. These findings suggest that the efficient utilization of biomass resources can be realized by process improvement for cogeneration of ethanol and l-lactic acid under the concept of biological refining.

## Methods

### Raw materials

Corn stalk was harvested in Zibo, Shandong of China. The chopped stalk was dried at 80.00 °C to a constant weight and milled to about 850.00 μm using a laboratory grinder. The DCS was composed of 34.65% glucan, 21.37% xylan, 1.96% arabinan and 20.51% lignin. The DCS was further pretreated by 2.00% (w/v) NaOH at 118.00 °C for 1.33 h, and the solids loading was kept at 10.00% (w/v). The solid fraction obtained by filtration after pretreatment was washed with water until the pH value of the residue attained 7.00. The ATCS contained 45.32% glucan, 12.12% xylan, 0.80% arabinan and 8.40% lignin, and 612.80 g ATCS (dry weight) could be obtained from 1.00 kg DCS. Cellulase, which had a specific activity of 78.00 FPU mL^−1^ according to the manufacturers’ data, was used for the enzymatic hydrolysis of ATCS, and it was provided by Tianfeng Bioengineering Corporation (Hebei, China).

Yeast extract (YE), beef extract and soya peptone were purchased from Aobox Biotechnology Co., Ltd. (Beijing, China). Malt extract was provided by Lifa Long Chemical Technology Co., Ltd. (Tianjin, China). Corn steep liquor powder (CSLP) was purchased from Beijing Mannafeed International Group (Beijing, China). All other chemicals used were reagent grade.

### Strains and culture media

*Saccharomyces cerevisiae* M3013 was screened by the research group of Tan in Beijing University of Chemical Technology. One *B. coagulans* strain was isolated and designated as LA1507 as reported in the previous study [18]. Both strains were maintained at the Key Lab of Bioprocess of Beijing.

For *S. cerevisiae* M3013, the agar medium contained 20.00 g L^−1^ glucose, 3.00 g L^−1^ malt extract, 3.00 g L^−1^ YE, 5.00 g L^−1^ soy peptone and 20.00 g L^−1^ agar, and the medium for inoculum preparation consisted of 0.80 g L^−1^ sucrose, 20.00 g L^−1^ glucose, 5.00 g L^−1^ soy peptone and 3.00 g L^−1^ YE. To evaluate the capability of carbon source utilization, *S. cerevisiae* M3013 was precultured in the medium containing 50.00 g L^−1^ glucose, 50.00 g L^−1^ xylose, 15.00 g L^−1^ YE, 2.05 g L^−1^ MgSO_4_∙7H_2_O and 0.50 g L^−1^ KH_2_PO_4_. The fermentation medium based on corn stalk consisted of ATCS hydrolysates (containing 120.30 g L^−1^ glucose and 33.50 g L^−1^ xylose) and 20.00 g L^−1^ CSLP.

For *B. coagulans* LA1507, the media for agar slant and inoculum were prepared as reported in the previous work [36]. To evaluate the capability of xylose utilization, *B. coagulans* LA1507 was precultured in the medium containing 15.00 g L^−1^ YE, 1.00 g L^−1^ NH_4_SO_4_, 0.40 g L^−1^ KH_2_PO_4_, 0.30 g L^−1^ MgSO_4_∙7H_2_O and 2.50 g L^−1^ NaCl, moreover, the initial xylose concentration in aerobic stage was 10.00 g L^−1^, and 50.00 g L^−1^ xylose was added in the anaerobic stage for l-lactic acid production. To investigate the effect of ethanol on the cell growth of *B. coagulans* LA1507 and the production of l-lactic acid, different ethanol concentrations ranging from 30.00 to 60.00 g L^−1^ were adopted, and the medium also contained 30.00 g L^−1^ xylose and 10.00 g L^−1^ CSLP. The medium for l-lactic acid production based on corn stalk was composed of the residual liquid after ethanol fermentation (containing 30.00 g L^−1^ xylose) and 10.00 g L^−1^ CSLP.

The pH of all culture media in this study was adjusted to 6.25 using 1.00 mol L^−1^ HCl or 40.00% (w/w) NaOH before use. Agar slants, inoculums, preculture media and the medium based on corn stalk for ethanol fermentation were sterilized at 116.00 °C for 0.33 h. However, the l-lactic acid fermentation medium deriving from residual liquid after ethanol fermentation was not sterilized.

### Cultivation conditions

*Saccharomyces cerevisiae* M3013 and *B. coagulans* LA1507 were maintained on agar slants which were stored at 4.00 °C. Stock cultures were transferred monthly, and strains of M3013 and LA1507 were grown on slants at 28.00 °C and 50.00 °C, respectively.

Inoculum preparation was conducted in conical flasks with a working volume of 100.00 mL. The seed culture of *S. cerevisiae* M3013 was incubated at 28.00 °C for 20.00 h, and the rotation speed was 180.00 rpm. The temperature and rotation speed during inoculum preparation of *B. coagulans* LA1507 were maintained at 50.00 °C and 150.00 rpm, respectively, and the incubation time was 20.00 h. The incubation rate of both strains was 10.00% (v/v).

Both of *S. cerevisiae* M3013 and *B. coagulans* LA1507 were precultured to examine the capacity of carbon sources utilization. Batch fermentations were conducted in a 5-L fermentor (SGB-5L, Changzhou Sungod Biotechnology & Engineering Equipment Co., Ltd., Jiangsu, China) with a working volume of 2.00 L. Ethanol fermentation was conducted at 30.00 °C with an agitation speed of 120.00 rpm, and the pH was kept at 6.00 by 40.00% (w/w) NaOH. In the aerobic stage of l-lactic acid fermentation, the aeration rate and agitation speed were maintained at 2.00 vvm and 400.00 rpm, respectively, and an agitation speed of 260.00 rpm was adopted in the following anaerobic fermentation. A temperature of 50.00 °C was adopted and the pH was maintained at 6.25 by 33.00% (w/w) Ca(OH)_2_.

To investigate the effect of ethanol on the cell growth of *B. coagulans* LA1507 and the production of l-lactic acid, batch fermentations were conducted with different initial ethanol concentrations (30.00 g L^−1^, 40.00 g L^−1^, 50.00 g L^−1^ and 60.00 g L^−1^), and a 5 L fermentor (SGB-5L, Changzhou Sungod Biotechnology & Engineering Equipment Co., Ltd., Jiangsu, China) with a working volume of 1.00 L (containing 30.00 g L^−1^ xylose and 10.00 g L^−1^ CSLP) was used, while keeping all other conditions identical to those adopted in the preculture of *B. coagulans* LA1507. The ethanol removed by gas stripping was collected by a condenser soaked in liquid nitrogen.

### The fed-batch hydrolysis of ATCS

A fed-batch process was conducted in the enzymatic hydrolysis of ATCS, and a cellulase dose of 25.00 FPU g^−1^ ATCS was adopted. The enzymatic hydrolysis was conducted at 55.00 °C with an agitation speed of 150.00 rpm, and the pH was maintained at 5.00 by 36.00% HCl. A 7-L fermentor (BLBIO-7GJ, Bailun Biological Technology Co., Ltd., Shanghai, China) with a working volume of 5.00 L was used as hydrolysis tank, and the final loading of ATCS was 25.00% (w/v). Finally, solid residue was removed from the enzymatic hydrolysates by vacuum filtration, and the ATCS hydrolysates normally contained 120.30 g L^−1^ glucose and 33.50 g L^−1^ xylose.

### The hybrid process for co-generation of ethanol and l-lactic acid based on corn stalk

**Fig. 7:**
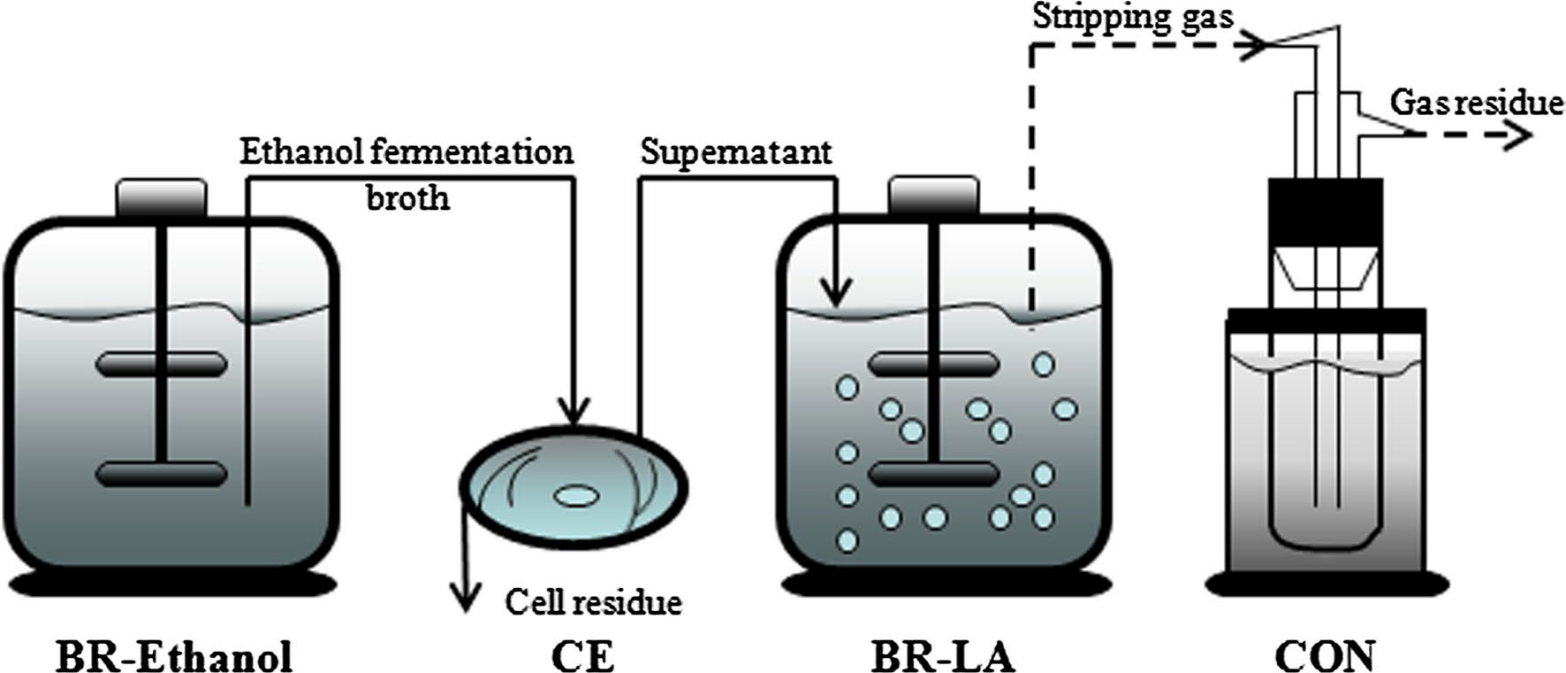
Schematic diagram of the experiments*. BR-ethanol* bioreactor for ethanol fermentation, *CE* centrifugation, *BR-LA* bioreactor for l-lactic acid fermentation, *CON* condenser using liquid nitrogen as coolant

The hybrid process involved in cogenerating ethanol and l-lactic acid is shown in Fig. 7. A 5-L fermentor (SGB-5L, Changzhou Sungod Biotechnology & Engineering Equipment Co., Ltd., Jiangsu, China) was use for ethanol and l-lactic acid fermentation. A working volume of 2.00 L was adopted in ethanol fermentation, and the fermentation broth obtained was centrifuged at 6275.00×*g* for 0.17 h to remove the cell residue. The supernatant (1.00 L) of ethanol fermentation broth was further used for l-lactic acid production. Particularly, the air pumped into the fermentor was used as both the carrier gas for single-pass gas stripping of ethanol and the oxygen provider for aerobic growth of *B. coagulans* LA1507, which could significantly reduce the energy cost of ethanol separation. All other conditions were identical to those adopted in the preculture of *S. cerevisiae* M3013 and *B. coagulans* LA1507. Liquid nitrogen was used as coolant to condense and recover the ethanol in stripping gas.

### Analytical methods

l-lactic acid was determined by SBA-40C biosensor analyzer (Institute of Biology, Shandong Province Academy of Sciences, Shandong, China) [37]. Before determination, insoluble substances were removed by centrifugation, and the samples were further diluted with distilled water. A spectrophotometer (UV-1100, Beijing Eternal Cause Instrument Co., Ltd., Beijing, China) was used to measure the cell density of *B. coagulans* LA1507 at a wavelength of 620.00 nm [38]. The yields of ethanol and l-lactic acid were calculated according to Eqs. (1) and (2), respectively:

1 $$Y_{\text{E}} = \frac{{W_{\text{ethanol}} }}{{W_{\text{glucose}} }} \times 100\% ;$$

2 $$Y_{\text{LA}} = \frac{{W_{\text{LA}} }}{{W_{\text{glucose}} + W_{\text{xylose}} }} \times 100\% ;$$

where *Y*_E_ and *Y*_LA_ represent the yields of ethanol and l-lactic acid based on carbon sources in this study. *W*_ethanol_ and *W*_LA_ are the total amount of ethanol and l-lactic acid produced, and *W*_glucose_ and *W*_xylose_ are the amount of glucose and xylose consumed in the fermentation. In this study, for batch fermentations of ethanol and l-lactic acid, the amount of glucose or xylose consumed was calculated according to Eq. (3):

3 $$W_{g/x} = C_{i} \cdot V_{i} - C_{f} \cdot V_{f} - \sum\limits_{1}^{n} {C_{t} } \cdot V_{t} ,$$

where *W*_*g/x*_ represents the amount of consumed glucose or xylose. *C*_*i*_ and *C*_*f*_ are the initial and final concentration of glucose or xylose, and *V*_*i*_ and *V*_*f*_ are the initial and final volume of fermentation broth. Particularly, *C*_*t*_ and *V*_*t*_ are the concentration and volume of a sample, and n represents the total sampling number.

The concentration of monosaccharides was determined by high-performance liquid chromatography (HPLC) equipped with a HPX-87P carbohydrate analysis column (Bio-Rad Labs, USA) and a refractive index (RI) detector, and the determination temperature was maintained at 80.00 °C using deionized water as mobile-phase (36.00 mL h^−1^) [39]. Ethanol concentration was analyzed based on the method of Cai et al. [40]. Moreover, for evaluation of mass balance in the experiment process, the analytical methods and calculation basis of carbohydrates and lignin in the fiber and liquid phases were according to the National Renewable Energy Laboratory (NREL) protocol [41].

## Abbreviations

LAB: lactic acid bacteria
DCS: dry corn stalk
ATCS: alkali-treated corn stalk
YE: yeast extract
CSLP: corn steep liquor powder
BR-Ethanol: bioreactor for ethanol fermentation
CE: centrifugation
BR-LA: bioreactor for l-lactic acid fermentation
CON: condenser using liquid nitrogen as coolant
RI: refractive index
HPLC: high-performance liquid chromatography
NREL: National Renewable Energy Laboratory
